# Alternative statistical methods for estimating efficacy of interferon beta-1b for multiple sclerosis clinical trials

**DOI:** 10.1186/1471-2288-11-80

**Published:** 2011-05-26

**Authors:** Makiko N Mieno, Takuhiro Yamaguchi, Yasuo Ohashi

**Affiliations:** 1 Department of Medical Informatics, Center for Information, Jichi Medical University, Shimotsuke, Japan; 2 Department of Biostatistics, Tohoku University School of Medicine, Sendai, Japan; 3 Department of Biostatistics, School of Public Health, University of Tokyo, Bunkyo, Tokyo, Japan

## Abstract

**Background:**

In the randomized study of interferon beta-1b (IFN beta-1b) for multiple sclerosis (MS), it has usually been evaluated the simple annual relapse rate as the study endpoint. This study aimed to investigate the performance of various regression models using information regarding the time to each recurrent event and considering the MS specific data generation process, and to estimate the treatment effect of a MS clinical trial data.

**Methods:**

We conducted a simulation study with consideration of the pathological characteristics of MS, and applied alternative efficacy estimation methods to real clinical trial data, including 5 extended Cox regression models for time-to-event analysis, a Poisson regression model and a Poisson regression model with Generalized Estimating Equations (GEE). We adjusted for other important covariates that may have affected the outcome.

**Results:**

We compared the simulation results for each model. The hazard ratios of real data were estimated for each model including the effects of other covariates. The results (hazard ratios of high-dose to low-dose) of all models were approximately 0.7 (range, 0.613 - 0.769), whereas the annual relapse rate ratio was 0.714.

**Conclusions:**

The precision of the treatment estimation was increased by application of the alternative models. This suggests that the use of alternative models that include recurrence event data may provide better analyses.

## Background

Multiple sclerosis (MS) is the most common demyelinating disorder of the central nervous system, and is characterized by repeated episodes of neurological dysfunction with variable remission. Since 1993, the beneficial effects of interferon beta have been shown [1], and in Japan, interferon beta-1b (IFN beta-1b) has significantly reduced relapse rates and reduced MRI lesion areas in patients with relapsing-remitting MS [2]. Recently, Kappos et al. [3] reported that IFN beta-1b can delay the conversion to clinically definite MS. Carroll [4] performed a comprehensive review of clinical studies of MS therapies.

The long-term treatment effects for chronic recurrent diseases such as MS should be evaluated in clinical trials. In the past, the primary endpoint in clinical trials of MS has been the annual relapse rate, the change in a clinical indicator such as the Expanded Disability Status Scale (EDSS) score or total area of MS lesions on the MRI scan from entry time, the proportion of non-relapsed patients, or the time to the first recurrence [1,2,5-10]. Meanwhile, extended methods of survival analysis for time-to-event data have been proposed, and such methods are useful when study subjects experience 2 or more events. Considering the recurrent events in survival analysis should theoretically increase the estimation efficiency regarding the effects of treatment [11]. Although these methods have not generally been applied to MS clinical trial data, Wang et al. [12] recently examined some of the models. Excellent reviews are available regarding how these methods can contribute to the estimation of treatment effects [11,13,14]. When using these models, it is important to pay attention to the nature of the models because the results of the estimation are highly dependent on the clinical situation [15]. In real clinical studies, the concerned events might occur rarely, several events might occur simultaneously, or several events might occur separately with high correlation. Appropriate models should be selected after considering the relationship between the assumptions of the models and the manner in which the events occur. For example, if we analyse the disease data such that the deteriorations of many lesions are found simultaneously, we should select the model that can manage the count data approach rather than the gap time modeling of event history analysis.

In this study, we focused on the extended Cox proportional models and Poisson regression model using Generalized Estimating Equations (GEE), which can be analyzed using existing statistical packages such as SAS. Using these regression models, we can estimate the adjusted treatment effect while considering the important covariates that might affect the outcomes, whereas the relapse rates provide only non-adjusted estimate [16]. The objective of this study was to investigate the performance of these models through a simulation study with MS-specific data generation processes and to apply various models that are used for estimating the treatment effect to a real clinical trial data set. This data set comprises the effect of IFN beta-1b on MS with special attention to subjects with relapsing-remitting MS.

## Methods

### Subjects

A phase II randomized controlled clinical trial was conducted to compare the effect of 2 different doses (high-dose: 250 μg and low-dose: 50 μg) of IFN beta-1b on relapsing-remitting MS in Japan. Details of the trial design, inclusion criteria, baseline demographics, and efficacy results have been published [2]. In the trial, 205 patients with relapsing-remitting MS were randomized, and efficacy was assessed in 188 patients (55 male and 133 female patients). The primary endpoint of the study was the evaluation of the annual relapse rate. The percentage of patients who experienced a relapse more than once during follow-up was 55.8% (53/95) of patients in the high-dose group and 65.6% (61/93) of patients in the low-dose group. In these groups, the maximum number of relapses was 7, and the minimum,- 0, with a median of 1. The annual relapse rates in the high- and low-dose groups as estimated by the person-time method were 0.763 and 1.069, respectively (relapse rate ratio = 0.714; 95% CI 0.560- 0.910; p = 0.006).

### Models

**Figure 1:**
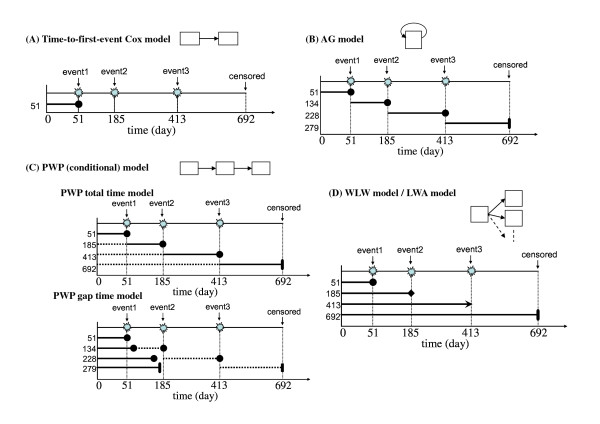
**Schematic forms of the various extended time-to-event Cox regression models**. Each arrow represents a stratum. Arrow diagrams describe about the behavior of ID = 1 in the sample data (DATA = MS) in Appendix (Additional file 1), who experiences the first event at day 51, second event at day 185, third event at day 413 and finally have censored at day 692. (A) "Time-to-first-event Cox model" only uses the information of the time to first event (the day 51). (B) "AG model" shows the renewal process of the events. (C) "PWP model" shows how the conditional models are constructed. (D) "WLW model" and "LWA model" model the marginal distribution of each event occurrence time.

Various survival models used for analysis of recurrent event data and that handle clustered and multiple event data have been proposed. Let *λ*_*ij*_(*t*) be the hazard function of the *j*th recurrence of the *i*th subject at time *t*; *λ*_0 *j*_(*t*) be the baseline hazard function of the *j*th recurrence at time *t*; *Y*_*ij*_(*t*) be the indicator variable for the *j*th recurrence of the *i*th subject at time *t*, which is 1 when the subject is at risk and under observation and 0 otherwise; X_*ij*_(*t*) be the *j*th covariate vector of the *i*th subject at time *t*; and β*j *be the parameter vector for the *j*th recurrence, which includes the treatment effect. When each recurrence is assumed to have common effect, we omit the subscript *j*. Schematic forms of the models are shown in Figure 1.

The first model to be considered is the ordinary time-to-first-event model, which is formulated with the Cox proportional hazard model (hereafter referred to as "time-to-first-event Cox model"). It handles only the time-to-first-event data and ignores the information of the second or more events. Hereafter, the models that can deal with this lack of information are shown.

Andersen and Gill [17] extended the Cox proportional hazard model in the counting process formation (AG model). A Poisson process in which, each counting process has independent increments is assumed so that multiple events within the same subjects are regarded independently. The hazard of subject *i *at time *t *is

Although subjects who have once experienced an event are excluded from the risk set from that time in the usual Cox model, subjects who have experienced at least 1 event and are under observation can also be included in the risk set in the AG model. Because the baseline hazard is assumed to be common among subjects, this model ignores the individual differences and might be effective when the overall treatment effects are of interest.

Prentice, Williams, and Peterson [18] also extended the Cox model. They proposed the conditional model, which assumes that a subject is not at risk for the *j*th event until he/she has experienced the (*j*-1)th event, where *Y*_*ij*_(*t*) is 0 until the (*j*-1)th event and after which it becomes 1 (PWP model). In terms of the time scale, 2 models are used. One model measures from the entry time and is called the total time model (PWP-T model). The hazard of the *j*th recurrence of subject *i *at time *t *is

The other model resets the clock at every recurrence and is called the gap time model (PWP-G model). Assigning *t*_*j*-1 _as the time at which the (*j*-1)th event occurs, the hazard of the *j*th recurrence of subject *i *at time *t *is

Although the PWP model makes the interpretation easy, the sizes of the risk sets become relatively small as the number of events increases, making the estimates unstable.

Wei, Lin, and Weissfeld [19] modeled the marginal distribution of the time of each occurrence of the event using the Cox model (WLW model). The hazard of the *j*th recurrence of subject *i *at time *t *is

In this model, each recurrence is modeled as a separate stratum, and each subject appears in all of the strata so that no assumptions are made with respect to the recurrence process. However, this may result in substantial efficiency loss because it ignores the obvious dependency structure, in that the (*j*+1)th recurrent time must exceed the *j*th.

On the other hand, Lee, Wei, and Amato [20] proposed a model (LWA model) that assumes a common baseline hazard, where the hazard can be written as

The same subjects can enter several risk sets simultaneously, although its unnaturalness is discussed at the same time.

In terms of the inference of the parameter vector, the use of robust variance, which can handle intra-subject correlations, is considered to be desirable for all models described above (AG, PWP, WLW, and LWA models). Regarding the parameter estimation of the PWP, WLW, and LWA models, each recurrence is assumed to have a common effect in this study.

The Poisson regression model fits the framework of the generalized linear models in which, the response variable, which is the number of occurrences of the event in a fixed time interval, follows Poisson distribution. Let *μ*(**X**) be the expected value of the number of relapses; *N*(**X**), the total observation period; *λ*(X), the constant relapse rate of MS; X, the covariate vector; and β, the parameter vector to be estimated. The relapse rate can then be written as

Thus, *μ*(**X**) = *N*(**X**)exp(**X'β**).

The relapse rate is not necessarily constant throughout the observation period; it is better to partition the time axis into intervals of constant rates.

Consequently, the intra-subject correlation of the relapse rate among the intervals can be discussed in terms of GEE. GEE is an extension of generalized linear models and regards a subject as a cluster so that the treatment effect can be estimated considering the correlation structure among response variables [21]. It is expected to be a flexible method for analyzing recurrent event data because it can be used even if many of the aforementioned assumptions regarding the proportional hazard models do not hold. In this study, the GEE-Poisson model was applied, and intervals were set at 6 months, each with the common rate.

### Simulation study

To determine which model is the most suitable for analyzing MS clinical trial data, we conducted the simulation study with consideration of the disease progression process or natural history. When performing a simulation study, we should examine the event generation process, which might be suited to the situation of the disease progression process [22]. The data generation process of this study was as follows. In a hypothetical randomized controlled clinical trial with placebo (n = 100) and active (n = 100) groups, we assumed that each patient had 10 hypothetical latent lesions in their brain and that the lesions were in the inactive phase at the entry time. The recurrence time was recorded after each lesion developed to the active phase. This setting modeled some MS pathological characteristics, such as time and spatial distribution of the lesions. The total follow-up period was set to 3 years, and the censoring time, which was assumed to be independent from the recurrence time, was generated using a Weibull distribution *S*(*t*) = exp (-*λt*^*γ*^) with the shape parameter γ = 2.1399 and the scale parameter λ = 0.000000576. The time to recurrence was also generated using a Weibull distribution, and 2 different scenarios were considered.

*Scenario 1*: All patients have individual identical Weibull distribution parameters, γ = 1.1452 and λ = 0.00141.

*Scenario 2*: Mixture population of 3 different sets of parameters; 46% of the population has γ = 1.2442 and λ = 0.000604, 45% of the population has γ = 1.1550 and λ = 0.001578, and 9% of the population has γ = 1.9694 and λ = 0.0000661.

The parameters used in our simulation study were calculated from other clinical trial data in Japan [23], especially for the placebo group, which can be regarded as the natural history cohort [24]. The mixture proportions described in Scenario 2 were obtained from the distribution of the number of recurrences in the year preceding the study, which was one of the important covariates. The hazard ratio (relapse rate ratio) of the active group to the placebo group was set at 1/1.3, such the true value of the Cox regression parameter (log-hazard ratio) was log(1/1.3) = -0.26236. Each simulation was repeated 1000 times, and the results were evaluated via the bias and mean square error (MSE). The bias is the difference between the estimated and true (or reference) values; thus, the treatment effect would be underestimated if we obtained positive bias and, overestimated if we obtained negative bias. The MSE considers both bias and variability as gauged by the variance of parameter estimates.

After the simulation study, the models were applied to the IFN beta real clinical trial data separately. All statistical analyses, including the simulation study, were performed using SAS version 9.1.3 (SAS Institute Inc., Cary, NC, USA). The SAS sample programs for the use of these models are shown in the appendix of this article (Additional file 1), and a dummy data set is used to clarify the use of the models.

## Results

The results of the simulation study are presented in Table 1. AG, Poisson, and GEE-Poisson models showed similar results, with positive bias and relatively small MSE in both scenarios. Almost no bias was detected in PWP-T, PWP-G, and LWA models in Scenario 1, whereas they showed larger bias in Scenario 2. In the WLW model, a relatively large bias with a negative direction was noted, indicating that an overestimation of the treatment effect and a large MSE were detected in both scenarios.

**Table 1:** Bias and MSE from the simulation study

	[Scenario 1]		[Scenario 2]	
Models	Bias	MSE	Bias	MSE
1: Time-to-first-event Cox regression	-0.002	0.049	0.023	0.046
2: AG model	0.044	0.014	0.090	0.030
3-1: PWP-T model	-0.001	0.018	0.080	0.022
3-2: PWP-G model	0.007	0.017	0.101	0.029
4: WLW model	-0.162	0.076	-0.064	0.064
5: LWA model	0.001	0.017	0.046	0.037
6: Poisson regression model	0.044	0.016	0.090	0.026
7: GEE-Poisson model	0.046	0.014	0.088	0.030

The various aforementioned models were then applied to the real clinical trial data introduced in the Methods section with adjustment for some important covariates, such as sex, age, EDSS score at entry time, total area of MS lesions on the MRI scan at the entry time, and number of recurrences in the year preceding the study. Table 2 shows the results of the analysis. The hazard ratio indicates the relative risk of the high-dose group to the low-dose group, and it is distributed from 0.613 (WLW model) to 0.769 (time-to-first-event Cox model). All models except the time-to-first-event Cox model showed a significant effect of high-dose IFN beta-1b. The standard error of the WLW model was the largest, while the PWP-T and PWP-G models showed relatively small values. The width of the confidence intervals of the AG, PWP-T, PWP-G, WLW, LWA, Poisson, and GEE-Poisson models was smaller than that of the time-to-first-event Cox model.

**Table 2:** Estimates of treatment effects for MS clinical trials in Japan

Models	Parameter Estimates	Standard Error	Hazard Ratio [95%CI]	P value
1: Time-to-first-event Cox regression	-0.263	0.194	0.769 [0.526, 1.123]	0.174
2: AG model	-0.377	0.170	0.686 [0.492, 0.957]	0.027
3-1: PWP-T model	-0.268	0.132	0.765 [0.591, 0.989]	0.041
3-2: PWP-G model	-0.306	0.135	0.736 [0.565, 0.960]	0.024
4: WLW model	-0.489	0.231	0.613 [0.390, 0.965]	0.035
5: LWA model	-0.427	0.195	0.653 [0.445, 0.957]	0.029
6: Poisson regression model	-0.371	0.171	0.690 [0.493, 0.965]	0.030
7: GEE-Poisson model	-0.352	0.169	0.703 [0.505, 0.980]	0.037

Regarding the behavior of the other covariates besides the IFN beta-1b variable, "the number of recurrences in the year preceding the study" showed significant differences in all 8 models. As the number increased, the hazard of recurrence in the study increased (range of hazard ratio among the 8 models: 1.164-1.375).

## Discussion

Because MS is a heterogeneous disease with a variety of subtypes and transitional cases, it is not easy to evaluate drug efficacy. By conducting a simulation study and applying it to real clinical trial data, we examined various extended Cox regression models and a Poisson regression model using GEE, which can handle recurrent events - not only the number of recurrences or the time to the first event, but all recurrences that occurred during the follow-up period. With the use of the extended models, significant effects were detected and the importance of utilizing more than 1 recurrent time was suggested by our analyses.

From the simulation study results, treatment effect was relatively overestimated in the WLW model. The same tendency was observed in the analysis of the real data; the WLW model showed the smallest hazard ratio. This overestimation tendency might not be desirable, especially in confirmatory trials. The bias and MSE of the LWA model in Scenario 1 were small for homogeneous population because of the similarity of the assumption of the data generation process; however, in Scenario 2, both bias and MSE became larger to some extent for heterogeneous population. In terms of MSE of the PWP-T and PWP-G models, totally preferable results were obtained, but the bias differences between Scenarios 1 and 2 for each model were approximately 2 times larger than those of the other models; this finding suggests of possible unstable features in the PWP models. For the time-to-first-event Cox model, AG model, Poisson regression model, and GEE-Poisson model, no extreme differences were found.

We are then left to select the best model for our data. All models have their own assumptions and characteristics, and so, our decision must consider the nature and system of disease progression that we have analyzed in advance in order to make the correct choice. When we consider the pathological condition in MS, such as the time and spatial distribution of latent lesions, the LWA model seems to be reasonable because of the assumption of a common baseline hazard, which means that each latent lesion has the same risk of development. If we can assume that all subjects have the same number of lesions that can develop at the same risk, the LWA model becomes conceivable. In the same way, if we can assume that all subjects have the same number of lesions that can develop at different risks, the WLW model seems to be best fitted. However, such settings would be unrealistic. In addition, the precision of the estimates in the WLW and LWA models is relatively poor. As the number of lesions increases, the number of strata also increases, which might lead to unstable estimates.

If we assume that the independent increments for all events are even among subjects, then the AG model is reasonable; however, this assumption would be unnatural in this case. PWP models would involve a similar situation despite small standard errors. In fact, the martingale residuals, which enabled us to examine the increment dependency, showed negative slopes throughout the period. This suggests that the assumptions that the AG and PWP models required would not exactly hold. The estimates of the Poisson regression and GEE-Poisson models were quite similar, so the advantage of using the GEE-Poisson model was not entirely clear in our study. However, if we had had a longer follow-up period and more time intervals, the method that accounts for the intra-subject correlation structure among intervals would be the more attractive model.

Realistically speaking, the assumptions needed in the extended Cox regression models (AG, PWP, WLW, and LWA models) would be difficult to be completely examined because of the uncertainty of MS pathological and/or clinical deterioration mechanisms and that fact that no one can prove the correctness of these assumptions. The Poisson regression and GEE-Poisson models are free of such assumptions. Moreover, in terms of the advanced nature regarding consideration of intra-subject correlation for recurrences in the GEE-Poisson model, the GEE-Poisson model is preferred over the Poisson regression model. However, further study regarding the behavioral characterization among these models is still needed.

## Conclusions

Our results indicate that the use of alternative models that include recurrence event data, especially the GEE-Poisson model, may provide better analysis for estimating the treatment effect.

## Competing interests

The authors declare that they have no competing interests.

## Authors' contributions

MNM, TY and YO participated in the design of the study. MNM performed the statistical analysis and drafted the manuscript. All the authors read and approved the final manuscript.

## Pre-publication history

The pre-publication history for this paper can be accessed here:

http://www.biomedcentral.com/1471-2288/11/80/prepub

## Supplementary Material

Additional file 1 **Appendix: SAS programming codes (example)**. The SAS sample programs for the use of the regression models shown in this study are provided using a dummy data set. Click here for file
