# Supplementary material for: Alternative statistical methods for estimating efficacy of interferon beta-1b for multiple sclerosis clinical trials
Source: BMC Med Res Methodol. 2011 May 26;11:80. doi: 10.1186/1471-2288-11-80 (PMC3118202; doi:10.1186/1471-2288-11-80)
Supplement: Additional file 1 — Appendix: SAS programming codes (example). The SAS sample programs for the use of the regression models shown in this study are provided using a dummy data set. [file 1471-2288-11-80-S1.PDF]

Appendix: SAS programming codes (example)

Consider some situation when subject ID=1 (TRT=0 group) observed 692 days and experienced three recurrences, subject ID=2 (TRT=1 group) 701 days and no recurrence, and subject ID=3 (TRT=1 group) 536 days and one recurrence, respectively. Then the sample data set (DATA=MS) contains the first three recurrences for each subject, and each recurrence time was measured from the subject's entry time into the study. The data consist of the following 6 variables:

ID: subject's identification

TRT: treatment group (0=placebo and 1=IFN beta)

OBSDAY: follow-up time

REC1, REC2, REC3: times of the three potential recurrences. A subject with only one recurrence has missing values in REC2 and REC3.

DATA=MS

ID	TRT	OBSDAY	REC1	REC2	REC3
**1**	**0**	**692**	**51**	**185**	**413**
**2**	**1**	**701**	**.**	**.**	**.**
**3**	**1**	**536**	**196**	**.**	**.**

First, consider fitting the AG model. The counting process style of input is used in the PROC PHREG specification. Three observations are created for each patient, one for each of the three potential recurrences. Four other variables are needed in DATA=AG:

TYPE: recurrence number (with value k for the kth potential recurrence)

T1: time of the (k-1)th recurrence for TYPE=k, or the entry time 0 if TYPE=1, or the follow-up time if the (k-1)th recurrence does not occur

T2: time of the kth recurrence if TYPE=k or follow-up time if the kth recurrence does not occur

STATUS: event status of T2 (0=censored and 1=recurrence)

DATA=AG

ID	TRT	T1	T2	STATUS	TYPE
**1**	**0**	**0**	**51**	**1**	**1**
**1**	**0**	**51**	**185**	**1**	**2**
**1**	**0**	**185**	**413**	**1**	**3**
**1**	**0**	**413**	**692**	**0**	**4**
**2**	**1**	**0**	**701**	**0**	**1**
**3**	**1**	**0**	**196**	**1**	**1**
**3**	**1**	**196**	**536**	**0**	**2**

The following SAS statements fit the AG model with DATA=AG:

```
PROC   PHREG   data=AG   covs(aggregate);
  model   (T1, T2)*STATUS(0) = TRT / rl   ties=exact;
  id   ID;
run;
```

In this model, inference is based on the robust sandwich covariance estimate, which is requested by the COVS (AGGREGATE) option in the PROC PHREG statement.

Next, consider the conditional models of PWP. In the PWP models, the risk set for the (k+1)th recurrence is restricted to those subjects who have experienced the first k recurrences. The following statements fit the PWP total time model and PWP gap time model.

```
DATA   PWP;
  set   AG;
  gap = T2-T1;
RUN;
*total time model;
PROC   PHREG   data=PWP   covs(aggregate);
  model (T1, T2)*STATUS(0)=TRT / rl   ties=exact;
  strata TYPE;
  id   ID;
RUN;
*gap time model;
PROC   PHREG   data=PWP   covs(aggregate);
  model gap*STATUS(0)=TRT / rl   ties=exact;
  strata TYPE;
  id   ID;
RUN;
```

When fitting WLW model or LWA model, recurrence times are regarded as multivariate failure times and WLW/LWA fits Cox model to each of the component times and makes statistical inference of the regression parameters based on a robust sandwich covariance matrix estimate. The data set is made like the following style.

DATA=WLW

ID	TRT	T2	STATUS	TYPE
**1**	**0**	**51**	**1**	**1**
**1**	**0**	**185**	**1**	**2**
**1**	**0**	**413**	**1**	**3**
**2**	**1**	**701**	**0**	**1**
**2**	**1**	**701**	**0**	**2**
**2**	**1**	**701**	**0**	**3**
**3**	**1**	**196**	**1**	**1**
**3**	**1**	**536**	**0**	**2**
**3**	**1**	**536**	**0**	**3**

The following statements fit the WLW model.

```
PROC PHREG data=WLW covs(aggregate);
 model T2*STATUS(0)=TRT / rl ties=exact;
 strata TYPE;
 id ID;
RUN;
```

If baseline hazard is assumed to be common among all strata, LWA model can be applied.

```
PROC PHREG data=WLW covs(aggregate);
 model T2*STATUS(0)=TRT / rl ties=exact;
 id ID;
RUN;
```

Poisson regression model is also fitted to the sample data set DATA=MS with a bit modification. The new variable RECNUM means the recurrence number during the observation period for each subject, and LogTime means the natural logarithm of OBSDAY.

DATA=MSpoisson

ID	TRT	OBSDAY	RECNUM	LogTime
1	0	692	3	6.5396
2	1	701	0	6.5525
3	1	536	1	6.2841

```
PROC GENMOD data=MSpoisson;
  class TRT;
  model RECNUM = TRT / dist=poisson link=log offset=logtime type3;
RUN;
```

In addition to the ordinal Poisson regression model, Poisson model with GEE can be fitted. When assuming the constant recurrence rate for each 180 days, the data set takes following style and we can fit the model with the statements below.

DATA=GEEPoisson

ID	TRT	time	RECNUM
1	0	180	1
1	0	180	1

**1**	**0**	**180**	**1**
**1**	**0**	**152**	**0**
**2**	**1**	**180**	**0**
**2**	**1**	**180**	**0**
**2**	**1**	**180**	**0**
**2**	**1**	**161**	**0**
**3**	**1**	**180**	**0**
**3**	**1**	**180**	**1**
**3**	**1**	**176**	**0**

```
PROC  GENMOD  data=GEEPoisson;
  class  ID  TRT;
  model  RECNUM = TRT  / dist=poisson  link=log  offset=logtime  type3;
  repeated  subject=ID  / type=exch;
  estimate   ‘P vs. A’  TRT  -1    1     /   exp ;
RUN;
```
